# Factors Related to Oral Health-Related Quality of Life of Independent Brazilian Elderly

**DOI:** 10.1155/2013/705047

**Published:** 2013-03-06

**Authors:** Karla Giovana Bavaresco Ulinski, Mariele Andrade do Nascimento, Arinilson Moreira Chaves Lima, Ana Raquel Benetti, Regina Célia Poli-Frederico, Karen Barros Parron Fernandes, Marina Lourdes Calvo Fracasso, Sandra Mara Maciel

**Affiliations:** ^1^Department of Preventive and Operative Dentistry, University of North Parana, Londrina, PR, Brazil; ^2^Department of Dentistry, State University of Maringa, Maringa, PR, Brazil; ^3^Institute of Odontology, University of Copenhagen, Copenhagen, Denmark

## Abstract

The aim of this cross-sectional study was to assess the factors associated with the impact of oral health on the quality of life in a sample of 504 Brazilian independent elderly. Data collection included oral examinations and structured interviews. The simplified form of the Oral Health Impact Profile (OHIP-14) was used to measure OHRQoL. Information on sociodemographic characteristics, use of dental services, and subjective measures of health was collected. Poisson regression within a hierarchical model was used to data analyses. The following variables were associated with a negative impact on OHRQoL: female gender (PR = 1.40; CI 95%: 1.11–1.77); lower class (PR = 1.58; CI 95%: 1.13–2.20); up to 3 occluding pairs of posterior teeth (PR = 1.88; CI 95%: 1.13–3.14); at least one untreated caries (PR = 1.28; CI 95%: 1.06–1.54); curative reasons for the last dental appointment (PR = 1.52; CI 95%: 1.15–2.00); poor self-perception of oral health (PR = 2.49; CI 95%: 1.92–3.24); and poor perception of dental care provided (PR = 1.34; CI 95%: 1.12–1.59). The younger elderly also noticed this negative impact. These findings showed that the clinical, sociodemographic, and subjective factors evaluated exerted a negative impact on OHRQoL in elderly people. Health authorities must address all these factors when planning interventions on oral health for this population.

## 1. Introduction

The health of the elderly is increasingly awakening the interest of researchers, since aging of the population, once seen as a phenomenon, is now a reality both in developed and developing countries [1]. It is estimated that by 2040 the developing countries will have 1 billion people aged 60 or over [2]. Given the great velocity and extent of this growth, care with this specific group is essential, so they can age healthily and with quality of life [3]. Oral health is among the factors that can exert influence on the quality of life of the elderly, since poor oral health conditions result in difficulty in chewing, speaking, or even in the relationships with other people [4–6].

In recent years a significant increase can be observed in studies on oral health-related quality of life (OHRQoL) in the elderly. The impact of oral health on quality of life broadens the sources of information from epidemiological research beyond just clinical indicators [7, 8]. Therefore, various instruments have been developed, including the Oral Health Impact Profile (OHIP-49), in order to measure people's perceptions of the social impact of oral disorders on their wellbeing. OHIP-49 was developed in Australia by Slade and Spencer [9]. Later, Slade [10] published a reduced version of this instrument, the OHIP-14. Although previous publications have used OHIP-14 to evaluate the influence of oral health on quality of life of the elderly [5, 11–13], only few studies have based their analysis using a theoretical hierarchical approach. 

Different models have been used to support the social determinants of health, among which the model proposed by Victora et al. [14] employs hierarchical frameworks to investigate the determinants of diseases by using multivariate analysis techniques. Although OHRQoL was not focused, that model has already been used in oral health studies and sustains the theory that sociodemographic conditions can determine oral morbidities and the use of health services, which in turn may have an influence on the elder's perception of his/her oral health [7, 15].

Considering that oral health might have a negative impact on the quality of life, the purpose of this study was to assess which factors are associated with this impact among elderly, living independently in the southern region of Brazil.

## 2. Methods

This cross-sectional study was approved by the Human Ethics Committee of the North Paraná University (pp.0070/09). The target population was comprised of independent elderly people, without physical and mental disabilities, aged 60 and over of both gender, from 38 primary healthcare centres in the urban region of Londrina, PR, Brazil. Other health indicators have been analyzed in this elderly population from the municipality, as part of a broader investigation conducted by the group of Interdisciplinary Aging and Longevity Study. It is worth noting that 85% of the elderly population commonly uses the Brazilian public health system in that city.

From a total of 43.610 individuals, a representative sample size was defined as 343 elderly, but 177 participants were added in order to compensate for sample loss (*N* = 520). Participants were randomly selected from information received from the primary healthcare centres. Stratified random sampling was used considering the city's five regions, obtaining 15% from the central region, 27% from the northern region, 23% from the southern region, 19% from the eastern region, and 16% from the western region. All participants signed an informed consent form.

Oral health was assessed through the experience and severity of dental caries (DMFT index), the presence of edentulism, and the use of and need for prosthesis according to criteria defined by the World Health Organization [16]. The dental exams were performed at the dental clinic of the University of North Parana, by a single examiner and intraexaminer agreement (kappa coefficient = 0.97) was considered excellent.

The instrument used to measure the impact of oral health on the quality of life of the elderly people was the OHIP-14 [10], which psychometric properties were previously validated in Portuguese [11]. The questionnaire is comprised of fourteen questions, corresponding to seven dimensions: functional limitation, pain, psychological discomfort, physical disability, psychological disability, social disability, and handicap. There are five possible answers for each question, according to the Likert-type scale: never, rarely, occasionally, frequently, and always. Answers were coded from 0 (never) to 4 (always), and OHIP-14 scores were calculated by the additive method. All the answers were added to produce a total score, which could vary between 0 and 56; whereby the higher the OHIP-14 score, the poorer the OHRQoL.

Initially, a descriptive analysis was made of the OHIP-14 results; thus, percentage distributions, mean, standard deviation, and median scores were obtained. Subsequently, the dependent variable was obtained by the dichotomization of the total OHIP-14 score, based on the median score (in this case 6). Therefore, all scores greater than 6 implied in having negative impact on OHRQoL, while all scores lower or equal to 6 represented not having negative impact on OHRQoL. The questionnaire was applied individually by two trained interviewers, and in addition to the OHRQoL interview (OHIP-14), other information was also collected. Variables were grouped from distal to proximal factors associated with *having negative impact in quality of life* in four blocks: block 1 = sociodemographic factors, block 2 = oral health conditions and needs, block 3 = use of dental services, and block 4 = subjective conditions, following a theoretical hierarchical model [14].

The following variables comprised the sociodemographic factors in block 1: gender (female/male); age (60–64 years, 65–74 years, 75 or over years); origin (rural area, urban area); companionship (none, with companionship); skin colour (white, nonwhite); schooling (up to 4 years, more than 4 years); economic class (upper, lower, according to the Brazilian Economic Classification criteria). 

The oral health conditions and needs in block 2 were comprised of number of teeth (edentulous, 1–19 teeth, ≥20 teeth); occluding pairs of posterior teeth (up to 3, more than 3); use of any type of prosthesis (yes, no); untreated caries lesions (none, at least one); need for any type of prosthesis (yes, no).

The use of dental services in block 3 was composed by visits to dental service (regular visits, not regular); last dental appointment (≤1 year, >1 year); type of dental service (public, private); reasons for seeking the last dental appointment (preventive, curative). 

The subjective conditions in block 4 were self-perception of overall health (very good/good, fair, and very poor/poor); self-perception of oral health (very good/good, fair, and very poor/poor); perception of dental care at the last appointment (good, poor). The questionnaire used was the same used in the last nationwide epidemiological survey carried out in Brazil with the purpose of obtaining a diagnosis of the population's oral health [17].

Poisson regression models with robust variance and adjustment for design effects were carried out using the Statistical Package for Social Science (SPSS), version 15.0. Bivariate analysis was performed at each hierarchical block. Results were presented as prevalence ratios (PR) with 95% confidence intervals (95% CI). In the multivariable analysis, variables were controlled for all others in the same block (horizontal), and those with a significant level of 5% or lower were retained to the next block down (vertical), thus characterizing the adjusted hierarchical model.

## 3. Results

Five hundred and four out of 520 elderly completed all aspects of the oral research (response rate of 96.9%). Regarding the sociodemographic factors (block 1), the average age was 69.5 years. The following characteristics were predominant: female gender (66.3%), white skin colour (62.1%), rural origin (53.8%), and with companionship (65.1%). The majority (80.5%) had schooling up to 4 years and belonged to the lower economic class (83.5%). 

When considering oral health conditions and needs (block 2), mean DMFT index was 26.1 (SD = 8.6): 87.1%, 8.9%, and 3.8% of the index accounted for the missing, filled, and decayed components, respectively. Almost half (47.4%) of the studied population was edentulous, and only 12.3% had ≥20 teeth. It is important to point out that 73.4% had no upper teeth, and 49.4% had no lower teeth. Regarding the use of and need for prosthesis, 60.3% used some type of prosthesis, 37.9% accounted for total prosthesis, and 54.5% needed full dentures in either the upper or lower jaw. 

In relation to the use of dental services (block 3), most of the participants did not visit the dentist regularly (95.4%) and had their last dental appointment >1 year (73.2%) in the private sector (77.2%) for curative reasons (93.8%). 

Regarding the subjective conditions (block 4), participants perceived their overall health as very good/good (38.1%), fair (47%), or very poor/poor (14.9%); their oral health as very good/good (40.5%), fair (25.6%), or very poor/poor (33.9%). Most perceived dental care at the last appointment as good (76.4%). 

Mean OHIP-14 scores were 9.1 (SD = 9.5), and median was 6.0. No negative impact of oral health on quality of life (score 0) was registered in 15.1% of the sample. A higher percentage of individuals reported to be concerned about their oral health and were uncomfortable to eat (Table 1). When considering the dimensions of OHIP-14, the highest means were registered for physical pain and psychological discomfort (Table 1).

Variables identified in the bivariate analysis (Table 2) that were associated to having negative impact on OHRQoL were for the 4 blocks: block 1—female gender, age 60–64 years, and lower economic class; block 2—up to 3 occluding pairs of posterior teeth, at least one untreated caries lesion; block 3—seeking the public dental service for curative reasons in the last dental appointment; block 4—very poor/poor self-perception of overall health and oral health, poor perception of dental care at the last appointment.

From the multivariate analysis (Table 3), the previously reported variables remained associated with negative impact on OHRQoL, with exception of site of last dental visit (block 3) and self-rating general status (block 4). 

## 4. Discussion

All the hierarchical blocks proposed in this study resulted in at least one significant variable associated with having negative impact on OHRQoL.

Among the sociodemographic variables, the female gender, the age between 60–64 years, and the lower economic class had negative impact on OHRQoL. When considering gender, women, even in similar clinical conditions to men, have been more unsatisfied with their appearance [18] and demonstrated greater perception of oral conditions [19–21] and higher complains regarding pain and the ability to chew [22]. 

Despite the evidence that oral health worsens with aging [23], in the present study, having negative impact on OHRQoL was inversely proportional to age, that is, the higher the age, the lower the OHIP-14 scores. A similar fact was also reported by McGrath and Bedi [24] and by Steele et al. [5], who observed that adults perceived a greater impact on OHRQoL than the elderly. Thus, it is presumed that the elderly become more tolerant towards oral health problems that result from aging [25].

The association between impact on OHRQoL and lower economic class of the elderly in this study is consistent with previous publications [10, 26]. This supports the fact that the less privileged class had less access to the dental services, despite their worse oral health condition [27]. As consequence, the accumulation and aggravation of oral problems may negatively impact on their quality of life [28]. Schooling, on the other hand, did not produce a high impact on OHRQoL in the population from this study, contrary to the reported by Atchison and Gift [29], who observed that individuals with lower schooling perceived a higher impact on OHRQoL.

In respect to the oral health conditions and needs, the high DMFT index and the high prevalence of edentulism observed in this study are similar to the ones reported in the latest epidemiological national survey [17] as well as other studies involving elderly Brazilians [30, 31]. These data reflect the Brazilian healthcare model, which historically has been centred on curative practices, often involving mutilation [32]. Surprisingly, the number of remaining teeth did not particularly influence the quality of life of the investigated elderly. The latest observation was also reported by Kim et al. [12] and may possibly be explained by the fact that tooth loss is associated with aging as a natural and inevitable process [29, 33]. This finding corroborates with previous reports that identified a positive perception from the majority of the elderly, in spite of their poor oral health [34, 35]. However, having fewer than three occluding pairs of posterior teeth had negative impact on OHRQoL, which is in accordance with previous findings [36–38] and can be explained by their importance to the masticatory ability [39]. Relationships between chewing ability and quality of life have been also found in elderly populations in countries such as Japan, Korea, and Great Britain [12, 39, 40]. The poor oral condition implies in greater treatment needs. During the validation of OHIP-14 in Brazil, significant higher treatment needs due to dental caries were associated with negative impact on OHRQoL [11], in agreement with the findings from this study. 

The need for and use of dental prosthesis among the elderly, on the other hand, were not associated with having negative impact on OHRQoL. Silva et al. [41] suggested that, although the absence of teeth and the use of deficient prosthesis do not interfere in daily activities or social relations, these conditions result in negative impacts on some of the OHIP dimensions, such as psychological discomfort, pain, and physical disability. In our study, higher scores in the dimensions “physical pain” and “psychological discomfort” were observed, similar to previous publications [12, 41, 42].

In this context, physical pain and psychological discomfort may exert a direct influence on the search for dental services. In the present study, pain or curative needs were the main reason related to the latest dental appointment and had negative impact on OHRQoL. This reinforces the idea that oral healthcare in Brazil has been based mainly on a curative approach. For many decades in the country, the oral healthcare has focused on school children, what consequently means that these assistance programmes excluded adults and the elderly who, for the most part, only received care in cases of dental emergencies [15]. It is also interesting to point out that only 26% of the elderly reported having used the public service for their last dental appointment, although all the participants from this study were registered in the public health system. The impact of the use of the public oral healthcare on OHRQoL was evident in the bivariate analysis, but this was not retained in the multivariate hierarchical analysis. Additionally, the elderly that reported poor service in their last dental appointment demonstrated higher OHIP-14 scores when compared to the ones who perceived the service as good. 

The subjective conditions also exert influence on OHRQoL, since the perception of individuals is more strongly influenced by self-evaluation than by objective parameters of their condition itself [43]. In this study, higher rates of negative impact on OHRQoL were found in elders with poor self-perception of oral health. Associations between the poor self-perception of oral health and its negative impact on OHRQoL have been well defined in the literature [11, 12, 41, 43]. 

The findings of the present study suggest that in order to assess and gain a better understanding on OHRQoL of elderly people, in addition to clinical conditions, sociodemographic profile and subjective conditions also have to be taken into consideration. 

Although the investigated population was representative of medium-sized municipalities, it should be noted that the sample was comprised of individuals registered in the primary healthcare centres. Therefore, the socioeconomic profile of the investigated population corresponds to the group of elderly who use the public health system in a specific southern municipality in Brazil. Considering the marked regional differences within Brazil, caution is recommended before generalizing the results from the present study. In addition, it is important to recall the cross-sectional nature of this study, and as such, it affords limitations that are typical of this kind of investigation. It is suggested that other surveys are carried out in order to establish a longitudinal perspective.

## 5. Conclusion

Clinical, sociodemographic, and subjective factors showed to have a negative impact on OHRQoL of the elderly people studied. Health authorities must address all these factors when planning interventions on oral health for this population.

## Figures and Tables

**Table 1:** Distribution of responses (%) to OHIP-14 items, mean and median scores subscales.

Conceptual domains and questions	Never (0)/hardly ever (1)	Occasionally (2)	Very often (3)/fairly often (4)	Mean (SD)	Median (range)
Functional limitation				1.34 (1.90)	0 (0–8)
Trouble pronouncing words	71.8	17.9	10.3		
Felt sense of taste worsened	82.8	8.1	9.1		
Physical pain				2.11 (2.19)	2 (0–8)
Had painful aching in mouth	70.3	17.6	12.1		
Uncomfortable to eat foods	58.7	20.0	21.3		
Psychological discomfort				2.02 (2.44)	1 (0–8)
Been self-conscious	57.4	19.0	23.6		
Felt tense	78.2	9.7	12.1		
Physical disability				1.37 (1.99)	0 (0–8)
Diet been unsatisfactory	73.0	12.7	14.3		
Had to interrupt meals	82.1	11.4	6.5		
Psychological disability				1.45 (2.06)	0 (0–8)
Difficult to relax	87.7	5.2	7.1		
Been a bit embarrassed	66.7	14.7	18.6		
Social disability				0.43 (1.17)	0 (0–8)
Irritable with other people	92.9	3.4	3.7		
Difficult doing usual jobs	93.6	3.6	2.8		
Handicap				0.39 (1.19)	0 (0–8)
Felt life less satisfying	90.8	4.0	5.2		
Totally unable to function	96.6	2.0	1.4		
Total OHIP-14				9.10 (9.47)	6 (0–56)

**Table 2:** Poisson bivariate analysis of factors associated with having negative impact on OHRQoL in Brazilian elderly (*n* = 504).

Variables	Having negative impact				
	Yes		Unadjusted PR	CI 95%	*P* value
	*n*	%			
*Block 1: sociodemographic factors *					
Gender					
Male	58	34.1	1		
Female	171	51.2	1.50	(1.18–1.89)	0.001
Age					
60–64 years	75	58.6	1		
65–74 years	113	41.4	0.70	(0.57–0.86)	0.001
75 or over	41	39.8	0.67	(0.51–0.90)	0.007
Geography					
Urban	108	46.4	1		
Rural	121	44.6	0.96	(0.79–1.16)	0.702
Living arrangements					
Not alone	147	44.8	1		
Alone	82	46.6	1.04	(0.85–1.26)	0.702
Ethnicity					
White	148	47.3	1		
Nonwhite	81	42.4	0.89	(0.73–1.09)	0.292
Education					
>4 years	42	42.9	1		
≤4 years	187	46.1	1.07	(0.83–1.38)	0.575
Economical class					
Upper/upper middle	25	30.1	1		
Lower middle/lower	204	48.5	1.60	(1.14–2.26)	0.006
*Block 2: oral health conditions and needs *					
Number of teeth					
≥20	24	38.7	1		
<1–19	103	50.7	1.31	(0.93–1.84)	0.120
Edentulous	102	42.7	1.10	(0.78–1.55)	0.580
Posterior occlusal pairs					
≥3	11	26.8	1		
<3	218	47.1	1.75	(1.04–2.93)	0.032
Use of prosthesis					
No	29	38.2	1		
Yes	200	46.7	1.22	(0.90–1.65)	0.191
Untreated dental caries					
No	139	42.1	1		
At least one	90	51.7	1.22	(1.01–1.49)	0.035
Prosthetic need					
No	44	40.4	1		
Yes	185	46.8	1.16	(0.90–1.49)	0.246
*Block 3: use of dental services *					
Pattern of dental attendance					
Regular	6	26.1	1		
Irregular	223	46.4	1.77	(0.89–3.56)	0.105
Time since last dental visit					
≤1 year	66	48.9	1		
1 year	163	44.2	0.90	(0.73–1.11)	0.337
Site of last dental visit					
Private/agreements	167	42.9	1		
Public	62	53.9	1.25	(1.02–1.54)	0.029
Reason for dental visit					
Checkup, exam, or clean	209	44.2	1		
Pain/problem	20	64.5	1.46	(1.10–1.93)	0.008
*Block 4: subjective conditions *					
Self-rating general status					
Good/very good	66	34.4	1		
Fair	122	51.5	1.59	(1.19–2.11)	0.001
Poor/very poor	41	54.7	1.49	(1.18–1.88)	0.001
Self-rating of oral health					
Good/very good	51	25.0	1		
Fair	52	40.3	1.61	(1.17–2.21)	0.003
Poor/very poor	126	73.7	2.94	(2.28–3.80)	0.000
Perception of the last query					
Good	154	40.0	1		
Poor	75	63.0	1.57	(1.31–1.89)	0.000

**Table 3:** Poisson multivariate analysis of factors associated with having negative impact on OHRQoL in Brazilian elderly (*n* = 504).

Variables	Adjusted PR	CI 95%	*P* value
*Block 1: sociodemographic factors *			
Gender			
Male	1		
Female	1.40	(1.11–1.77)	0.004
Age			
60–64 years	1		
65–74 years	0.72	(0.59–0.88)	0.001
75 or over	0.68	(0.52–0.90)	0.006
Economical class			
Upper/upper middle	1		
Lower middle/lower	1.58	(1.13–2.20)	0.008
*Block 2: oral health conditions and needs *			
Posterior occlusal pairs			
≥3	1		
<3	1.88	(1.13–3.14)	0.015
Untreated dental caries			
No	1		
At least one	1.28	(1.06–1.54)	0.010
*Block 3: use of dental services *			
Reason for dental visit			
Checkup, exam, or clean	1		
Pain/problem	1.52	(1.15–2.00)	0.003
*Block 4: subjective conditions *			
Self-rating of oral health			
Good/very good	1		
Fair	1.48	(1.08–2.02)	0.014
Poor/very poor	2.49	(1.92–3.24)	<0.001
Perception of the last query			
Good	1		
Poor	1.34	(1.12–1.59)	0.001
